# Quantitative analysis of mRNA translation in mammalian spermatogenic cells with sucrose and Nycodenz gradients

**DOI:** 10.1186/1477-7827-8-155

**Published:** 2010-12-25

**Authors:** Kenneth C Kleene, Jana Bagarova, Sabrina K Hawthorne, Leah M Catado

**Affiliations:** 1 Department of Biology, University of Massachusetts, Boston, MA 02125-3393, USA; 2 Cardiovascular Research Center, Massachusetts General Hospital, 50 Blossom Street, Boston, MA 02114, USA; 3 Upper School Science Department, Buckingham, Browne and Nichols School, 80 Gerry's Landing Road, Cambridge, MA 02138, USA

## Abstract

**Background:**

Developmental and global regulation of mRNA translation plays a major role in regulating gene expression in mammalian spermatogenic cells. Sucrose gradients are widely used to analyze mRNA translation. Unfortunately, the information from sucrose gradient experiments is often compromised by the absence of quantification and absorbance tracings, and confusion about the basic properties of sucrose gradients.

**Methods:**

The Additional Materials contain detailed protocols for the preparation and analysis of sucrose and Nycodenz gradients, obtaining absorbance tracings of sucrose gradients, aligning tracings and fractions, and extraction of equal proportions of RNA from all fractions.

**Results:**

The techniques described here have produced consistent measurements despite changes in personnel and minor variations in RNA extraction, gradient analysis, and mRNA quantification, and describes for the first time potential problems in using gradients to analyze mRNA translation in purified spermatogenic cells.

**Conclusions:**

Accurate quantification of the proportion of polysomal mRNA is useful in comparing translational activity at different developmental stages, different mRNAs, different techniques and different laboratories. The techniques described here are sufficiently accurate to elucidate the contributions of multiple regulatory elements of variable strength in regulating translation of the sperm mitochondria associated cysteine-rich protein (*Smcp*) mRNA in transgenic mice.

## Background

The regulation of mRNA translation plays an important role in gene expression in spermatogenic cells. The mRNAs encoding many proteins that are first synthesized during the final stages of sperm differentiation are transcribed in early spermatids, stored as translationally repressed free-mRNPs for up to a week in mice, and actively translated in late spermatids [1]. However, developmental regulation of mRNA translation applies to some mRNAs that are expressed in meiotic cells, notably the *Pgk2 *mRNA, and all mRNAs in meiotic and haploid spermatogenic cells are partially repressed by a global mechanism(s), some of which appear to undergo little developmental regulation [1-3].

Sucrose gradients are frequently used to study mRNA translation in spermatogenic cells, because the method can be used to study the translational activity of any mRNA for which probes can be designed. Although sucrose gradients have produced important insights into translational regulation in spermatogenic cells, this body of information is compromised for technical reasons. A common problem is that many sucrose gradient analyses lack absorbance tracings, which are necessary to establish that the translational activity of the cell population under analysis is normal. Another problem is that the polysome loading of mRNAs is rarely quantified. Quantification is necessary to compare polysome loading of different mRNAs, experimental protocols, developmental stages, and laboratories. Quantification has also provided evidence that translation of the sperm-mitochondria cysteine-rich protein mRNA (*Smcp*) 5' and 3'UTR contain multiple elements which regulate translation of the green fluorescent protein (GFP) coding region in transgenic mice to different extents [4]. Another problem is that the properties of sucrose gradients are not universally understood, leading to errors in interpretation.

The purpose of the present article is to describe in detail the techniques for sucrose gradient analysis, and quantification of polysome loading. We also describe a second type of gradient analysis of mRNA translation, Nycodenz gradients, which are not used nearly as frequently as sucrose gradients, but appear to have advantages in certain situations.

## Methods

The methods for the quantitative analysis of mRNA translation in Nycodenz and sucrose gradients in prepubertal and adult testis, purified spermatogenic cells and cultured seminiferous tubules have been published previously [4-9]. Additional file 1 contains detailed protocols for sucrose and Nycodenz gradient analysis, and Additional file 2, Additional file 3 and Additional file 4 (Figures S1, S2 and S3) illustrate techniques for pouring and collecting gradients.

## Results and discussion

The methods for estimating polysomal loading involve two main issues, identification and separation of free-mRNPs and polysomal mRNA, and quantification. These are discussed separately below.

### Separation and identification of free-mRNPs and polysomal mRNAs

The proportion of mRNA that is translationally active, polysomal loading, can be measured by analysis of sedimentation velocity in sucrose gradients and equilibrium density in Nycodenz gradients. The former is much more commonly used than the latter. Both types of gradients are based on the biophysical consequences of the fact that translationally active mRNAs, but not translationally inactive free-mRNPs, are associated with a number of ribosomes that is approximately proportional to the length of the coding region [5,10,11]. As a result, polysomal mRNAs sediment more rapidly than do free-mRNPs in sucrose gradients, because the rate of sedimentation of the polysomal mRNA is determined primarily by the number of ribosomes bound to the mRNA. In contrast, Nycodenz gradients separate polysomes and free-mRNPs by differences in density. Since the ratio of protein to RNA in ribosomes is lower than that in free-mRNPs, ribosomes and polysomes equilibrate at higher densities than do free-mRNPs [4,12-15]. Thus, Nycocenz gradients separate cytoplamic extracts into three major fractions: ribosomes and polysomes near the bottom, free-mRNPs in the middle, and proteins near the top. Sanz et al. [16] developed a third method of separating free-mRNPs and polysomal mRNAs which does not depend on the biophysical properties of free-mRNPs and polysomes: immunoprecipitation of polysomes in genetically engineered mice in which the natural gene encoding ribosomal protein L22 (RPL22) is replaced by a transgene in which RPL22 is tagged with the HA-epitope.

Sucrose gradients and Nycodenz gradients have complementary advantages. Sucrose gradients are superior to Nycodenz gradients in providing information about the sizes of free-mRNPs and polysomes. In general, the rate of sedimentation of free-mRNPs is correlated with the length of the mRNA. For example, the peaks of *Prm1 *(550 nt), *Smcp *(900 nt) and *Pabpc1 *(3000 nt) free-mRNPs sediment at 20S, 50S and 80S, respectively [5], and most active mRNAs exhibit peak polysome sizes that are directly correlated with the length of the coding region in sucrose gradients, although the spacing between ribosomes increases with the length of the coding region [5,10,11]. This is supported by observations that polysomes containing coding regions of variable length, *Prm1 *(153 nt), *Smcp *(429 nt), *Gfp *(720 nt) and *Ldhc *mRNAs (993 nt), sediment respectively with polysomes containing, 3-4, 3-4, 5-6 and 7-8 ribosomes/polysome [5,6]. In addition, sucrose gradients reveal regulatory mechanisms affecting polysome size, arguably the most important of which is upstream reading frames which sharply reduce polysome size [6,11,17]. By comparison, Nycodenz gradients are easier to prepare, don't require the specialized equipment needed for sucrose gradients (a gradient former and UV analyzer), and separate polysomes and free-mRNPs of most mRNA species into two discrete fractions with minimal effects of mRNA size [4].

**Figure 1:**
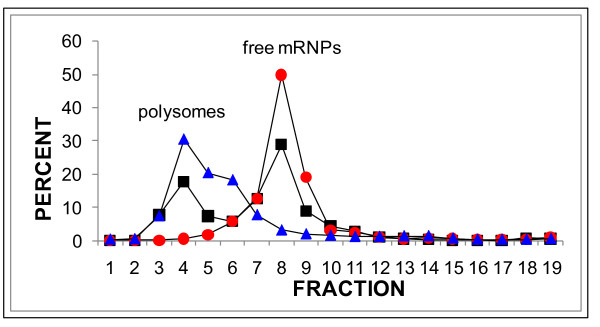
**Nycodenz gradient analysis of 18S ribosomal RNA, and the *Ldhc *and *Smcp *mRNAs in 26 dpp prepubertal mouse testis**. A cytoplasmic extract of the testes of three 26 dpp mice was sedimented on a Nycodenz gradient, collected as 19 fractions from the bottom, RNAs were extracted from each fraction, and the levels of 18S rRNA, *Ldhc *and *Smcp *mRNAs were determined by sequential hybridization of each probe to a Northern blot and phosphorimage analysis. The methods and probes are described in the Additional file 1 and [5]. 18s rRNA, blue triangles; *Ldhc *mRNA, black squares; *Smcp *mRNA, red circles. The peaks of polysomes and free-mRNPs are indicated.

Nycodenz gradients may also provide more accurate estimates of the levels of polysomal mRNA in cell populations in which mRNA translation is strongly repressed. Figure 1 depicts an experiment in which a cytoplasmic extract of 26 dpp testes was sedimented on a Nycodenz gradient and the distribution of the *Smcp *and *Ldhc *mRNAs and 18S ribosomal RNA was quantified by phosphorimage analysis of Northern blots. Note that all of the gradients in this study were collected manually from the bottom. Figure 1 shows that the peaks of *Smcp *and *Ldhc *free-mRNPs align in Fraction 10, that the peak of polysomal *Ldhc *mRNA aligns with the peak of 18S rRNA in fraction 4, and that the levels of *Smcp *mRNA increase continuously in fractions 4-6. Fraction 4 contains ~0.6% of the total *Smcp *mRNA on the gradient. The absence of a peak of *Smcp *mRNA in fraction 4, implies that the levels of polysomal *Smcp *mRNA are negligible at this age, in agreement with the observation that the most advanced spermatids in 26 dpp testis are ~step 9, and therefore lack the step 11-16 spermatids in which the SMCP protein is detected [18].

Several controls are commonly used to establish that mRNAs are associated with polysomes in sucrose gradients.

First, absorbance tracings identify regions of sucrose gradients containing mRNAs that sediment faster than monosomes, polysomes, and slower than monosomes, free-mRNPs. The relationship between coding region length and polysome size in sucrose gradients provides another argument supporting the identification of polysomes translating specific mRNAs [4,5]. Additional file 1 describes methods for obtaining absorbance tracings and aligning tracings and fractions of sucrose gradients. The positions of polysomes in Nycodenz gradients are usually established by agarose gel electrophoresis of ethidium bromide stained ribosomal RNA [4,12,13].

Second, analysis of the distribution of control mRNAs that sediment with free-mRNPs and/or polysomes. The *Prm1 *and *Prm2 *mRNAs are often used for this purpose because the length of these mRNAs correlates with translational activity: *Prm1 *and *Prm2 *mRNAs in free-mRNPs have long, homogenous poly(A) tails, while the polysomal mRNAs have heterogeneous, shortened poly(A) tails [7,16]. The *Ldhc *mRNA, encoding the testis-specific isoform of lactate dehydrogenase, is another useful control mRNA, because four laboratories report that it exhibits high and essentially constant levels of polysomal mRNA in pachytene spermatocytes, round spermatids, and prepubertal and and adult testis in sucrose gradients [4,19-21].

Third, mRNAs that sediment with polysomes in Mg^++^-containing sucrose gradients shift to the mRNP fractions after disruption of the associations of the ribosomal subunits with each other and with mRNA by EDTA. Somewhat surprisingly EDTA-treatment does not shift all polysomal mRNA to the mRNP fractions; a minor fraction of some mRNA species sediment with small polysomes [5].

These methods usually seem to work well. In most cases, inferences of translational activity based on expression of various testicular proteins, the proportions of mRNAs sedimenting with polysomes and free-mRNPs in sucrose and Nycodenz gradients, and dissociation by EDTA are in complete agreement [1,4].

However, the identification of polysomal mRNAs is complicated by several recent reports of translationally repressed mRNAs that sediment with polysomes in sucrose gradients. These large, inactive mRNPs include "pseudopolysomes" induced by microRNAs in *Drosophila *cell-free translation systems [22,23], and a transgenic mRNA containing the *Gfp *5'UTR, the *Gfp *coding region and the *Smcp *3'UTR in 21 dpp mouse testis [4]. 55% of this transgenic *Smcp-Gfp *mRNA sediments with polysomes in sucrose gradients, despite the fact that GFP fluorescence is undetectable and 3% of the mRNA equilibrates with polysomes in Nycodenz gradients [4]. In addition, ~21% of the *Ldhc *mRNA appears to be in large, inactive mRNPs based on findings that 56% and 35% is polysomal in sucrose and Nycodenz gradients, respectively, in both 21 dpp and adult testis [4]. The frequency of inactive mRNAs that form polysome-sized mRNPs in sucrose gradients in spermatogenic and somatic cells is not known.

Unfortunately, there are no simple tests that reliably distinguish between large inactive complexes and *bona fide *polysomes in sucrose gradients. The large, inactive complexes mentioned above are dissociated by EDTA [4,5,22], thus invalidating the most common control. In theory, inhibitors of protein synthesis that dissociate polysomes, such as pactamycin and puromycin, should be more specific than EDTA, but these inhibitors partially dissociate polysomes in somatic cells and spermatids ([24], Cataldo and Kleene, unpublished). It would take careful quantitative analysis of multiple gradients to make a convincing statistical argument that polysomes have been dissociated. The observation that 55% and 3% of the transgenic *Smcp-Gfp *mRNA mentioned above sediment with polysomes in sucrose and Nycodenz gradients, respectively, suggests that Nycodenz gradients may be a more reliable control than dissociation with EDTA to establish that a mRNA is associated with polysomes [4]. However, Wang et al. [23] report that pseudopolysomes contain large numbers of small ribosomal subunits without large subunits, and are therefore expected to equilibrate with polysomes in Nycodenz gradients. Immunoprecipitation of polysomes containing RPL22-HA should produce unambiguous results [16], but would require several generations of mouse breeding to replace the natural RPL22 gene with the homozygous RPL22-HA gene before transgenic mRNAs could be analyzed.

The *Prm1 *mRNA is the most commonly used control mRNA to establish the positions of free-mRNPs and polysomes in sucrose gradients. This mRNA has potential problems because its coding region is one of the smallest in mammalian cells, 153 nt. For example, an experiment in which polysomal *Prm1 *mRNA sediments near the bottom of a sucrose gradient is one in which large polysomes have pelleted. Thus, failure to analyze the mRNA in the pellet could lead to the erroneous conclusion that a large mRNA is translationally inactive. We know of no evidence that the common practice of placing a 60% sucrose pad on the bottom of sucrose gradients effectively prevents large polysomes from pelleting.

Differences in the sizes of mRNPs and polysomes containing mRNAs of varying sizes also create difficulties in dividing sucrose gradients into two fractions (free-mRNPs and polysomal mRNAs) for microarray studies of global mRNA translation.

The small size of the *Prm1 *and *Prm2 *mRNAs may create another potential problem. For many years it was generally believed that the number of ribosomes associated with specific mRNAs is usually proportional to the length of the coding region and that the ribosomes are spaced ~100 nt apart on coding regions [10]. Arava et al. [11,25] demonstrate that this rule does not apply in yeast: mRNAs with short coding regions have more ribosomes bound per 100 nt than do mRNAs with longer coding regions, apparently due to more rapid initiation. Arava et al. [11] extend this conclusion to mouse testis by analyzing sucrose gradient data reported by Cataldo et al. [5]. Before Arava et al. [11] it seemed reasonable to assume that the distribution of the *Prm1 *mRNA in sucrose gradients accurately reflects the translation of all mRNAs. Now, it is unclear whether this is true: the size and levels of *Prm1 *polysomes may be less sensitive to stress than those of mRNAs with longer coding regions.

Another misconception is that the all of the proteins detected by Western blots that sediment slower than single ribosomes are in free-mRNPs. In reality, most free-proteins sediment between 2 and 7S in sucrose gradients [26], while most free-mRNPs sediment between 20-80S [5]. The distinction is critical: the constituents of free-mRNPs conceivably function as translational repressors, while free-proteins cannot.

### Quantification of polysome loading

Accurate quantification of polysomal mRNA requires attention to details in the preparation of the cytoplasmic extract for the gradients, extraction of RNA from the gradient fractions, and quantification of mRNA levels.

Quantitative analysis of mRNA translation in sucrose gradients should begin with absorbance tracings which demonstrate normal levels of polysomes for testis. Four laboratories have reported similar absorbance tracings of extracts of adult testis with a major peak of 80S single ribosomes and a broad hump of polysomes with maximum absorbance in polysomes containing 5-7 ribosomes/polysome [4-6,9,27-29]. Additional file 3, Figure S2, displays an absorbance tracing of 21 dpp testis which is indistinguishable from those of adult testis except that the absorbance peak of the 60S ribosomal subunit is consistently higher than that of 80S ribosomes [4,6]. The opposite is true of adult testis. Unfortunately, many sucrose gradient analyses of total testes and virtually all analyses of purified spermatogenic cells lack absorbance tracings, leaving questions unanswered about the health of the cell population that was analyzed.

The distribution of a control mRNA in sucrose gradients is often used to argue that translation is normal. However, this argument is invalid in the absence of quantification: some sucrose and Nycodenz gradients have been "validated" with control mRNAs that exhibit virtually no polysomal mRNA.

**Figure 2:**
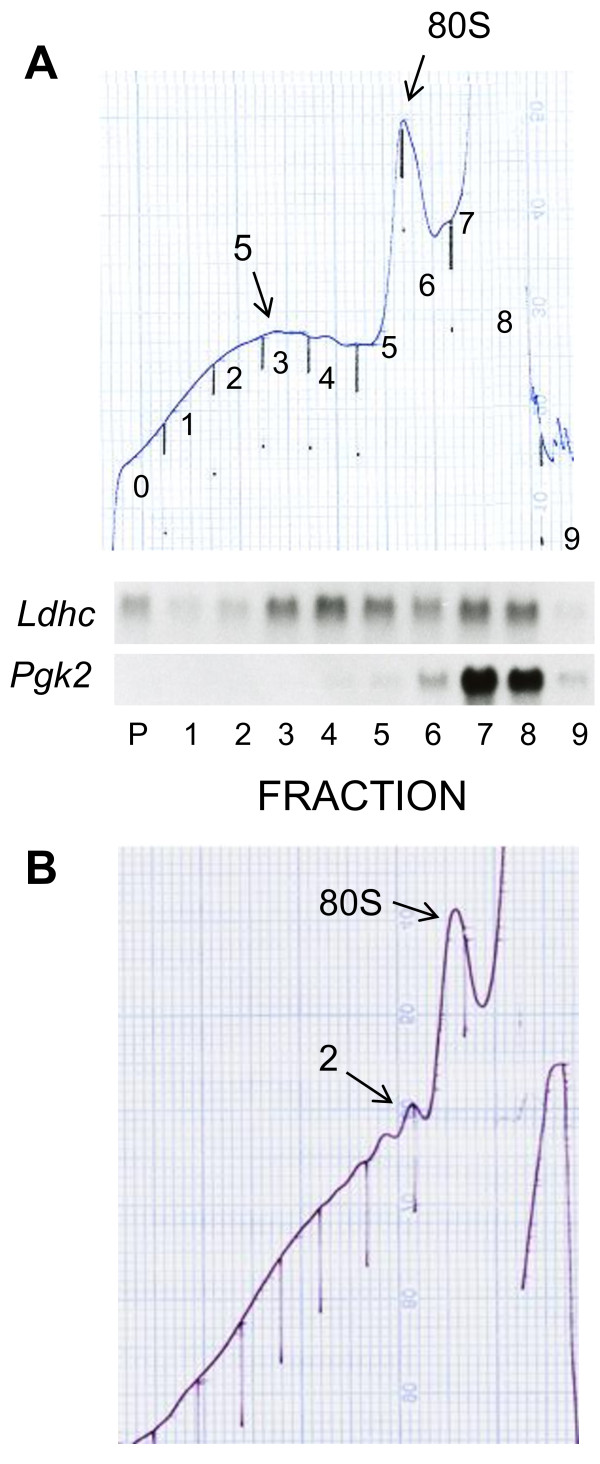
**Analysis of mRNA translation in purified pachytene spermatocytes purified by sedimentation on bovine serum albumin gradients**. Dissociated testicular cells from 16 dpp (A) and adult mice (B) were purified by sedimentation at 1XG on bovine serum albumin gradients, pachytene spermatocytes were collected, cultured for 1 hr at 32°C in RPMI 1040 medium in 5% CO_2 _in air, cytoplasmic extracts were prepared and sedimented on sucrose gradients. The preparation in panel A contained 4.28 × 10^6 ^pachytene spermatocytes. The gradients in panels A and B were sedimented at 35,000 rpm in the SW60Ti rotor for 100 and 80 min, respectively. The absorbance tracings of both gradients at 254 nm are shown. In addition, in Panel A the RNAs were extracted from each fraction, and the distribution of the *Ldhc *and *Pgk2 *mRNAs was analyzed by sequential hybridization of a single Northern blot. The fractions were collected from the bottom, and the fraction labeled P contains RNA extracted from the pellet on the bottom of the ultracentrifuge tube. The full scale absorbance of the UV analyzer was 0.32 using a flow cell with a 2 mm path length. The positions of the fractions in the absorbance tracing in panel B are numbered. Fraction 0 in the absorbance tracing is the void and does not contain RNA.

Another problem is illustrated in Figure 2, which displays the absorbance tracings of two sucrose gradients of pachytene spermatocytes that were purified by sedimentation of single cell suspensions of seminiferous tubules at unit gravity on bovine serum albumin gradients. Figure 2 demonstrates that ~4 × 10^6 ^pachytene spermatocytes contain sufficient ribosomes to obtain absorbance tracings. In addition, the tracing in Figure 2B exhibits drastically reduced polysomes, while the tracing in Figure 2A exhibits a more modest reduction in polysome size demonstrated by the smaller amount of large polysomes compared with those in total testis and seminferous tubules [4-6,9,27-29].

The Northern blots in Panel A demonstrate that the levels of the *Ldhc *mRNA that sediment with polysomes, ~60%, are similar to those in freshly dissected adult and prepubertal testis, ~56% (Table 1 and [4,5]). However, the size of polysomes translating the *Ldhc *mRNA is reduced based on differences in the number of fractions separating free-mRNPs and polysomes in purified cells and freshly dissected testis, 1 vs. 3-4 [4]. The Northern blot also demonstrates that polysomal *Pgk2 *mRNA is undetectable in pachytene spermatocytes purified from 16 day prepubertal testes, an age which contains no round spermatids [30]. We have previously demonstrated that ~33% of the *Pgk2 *mRNA sediments with polysomes in adult testis [5]. In combination, these findings demonstrate that the *Pgk2 *mRNA is repressed in pachytene spermatocytes and translated in spermatids.

**Table 1:** Quantification of polysomal loading of Ldhc and Smcp mRNAs at different stages by different methods

**mRNA(Age)**^**1**^	**Nycodenz**^**2 **^**Phos**^**3**^	**Nycodenz qPCR**^**3**^	**Sucrose**^**2 **^**Phos**	Sucrose qPCR	**Immuno**^**2 **^**Phos**
*LdhC *(21 dpp)	33.4, 36.0^4^	34.6 ± 3.2 (6)	57.7, 58.1	51.0 ± 9.3 (6)	^5^ND
*LdhC *(Adult)	32.6 ± 2.7 (3)	28.4	56.3 ± 2.1 (9)	56.8 ± 2.7 (5)	ND
*Smcp *(21 dpp)	2.2 ± 0.6 (3)	3.0, 4.3	6.7, 8.3	3.7 ± 2.8 (4)	ND
*Smcp *(Adult)	32.8 ± 3.2 (3)	33.8	35.3 ± 4.9 (11)	29.8 ± 9.3 (4)	ND
*Prm1 *(25 dpp)	ND	ND	ND	ND	9.8 ± 1.9 (3)
*Prm1 *(32 dpp)	ND	ND	ND	ND	29.8 ± 3.7 (3)
*Prm1 *(Adult)	ND	ND	24.5 ± 6.5 (3)	ND	ND

^1^Name of species of mRNA and (age of testes analyzed)

^2^Method of separation of free-mRNPs and polysomal mRNAs: sucrose gradient, Nycodenz gradient, immunoprecipitation of HA-epitope tagged RP22 [16]

^3^Method of quantification of levels of mRNA: phosphorimage of Northern blots or slot blots or RT-qPCR

^4^Percent of mRNA associated with polysomes. The results are expressed and mean and standard deviation with number of replicate determinations in parentheses. The absolute values are given when there one or two replicates. The values are derived from references [4-6,16].

^5^ND, not determined

We do not know how to avoid the artifactually small polysomes in purified spermatogenic cells. The breakdown is presumably caused by cellular damage and/or stress during dissociation and cell separation, possibly including disruption of the associations of spermatogenic cells and Sertoli cells, because the absorbance tracings of sucrose gradients of seminiferous tubules that have been cultured for one hour are identical to those of freshly dissected adult testis [8,9]. Culturing purified spermatogenic cells in medium containing low levels (0.25 μg/ml) of cycloheximide for one hour, a remedy for low efficiency of translation initiation [8], does not restore the size of the polysomes to those of freshly dissected testes and cultured seminiferous tubules (Kleene, unpublished).

**Figure 3:**
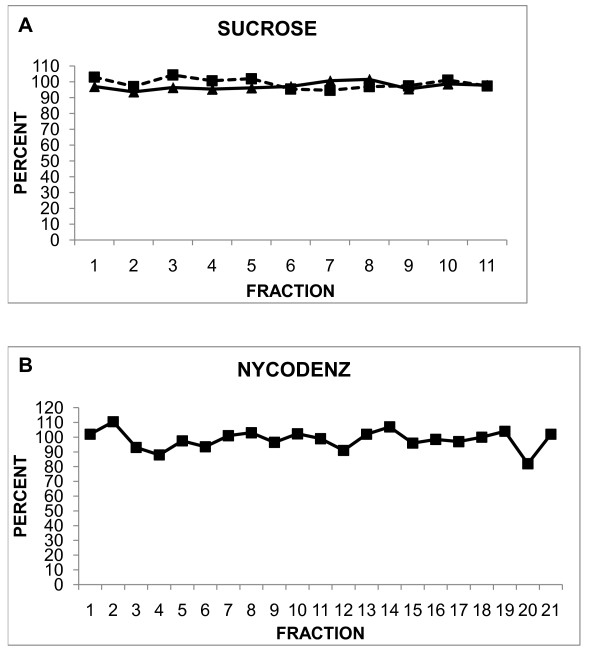
**Recovery of [^32^P]-labeled T7 bacteriophage RNA polymerase transcripts from the fractions of sucrose and Nycodenz gradients**. Each fraction of sucrose and Nycodenz gradients was spiked with ~100,000 cpm of [^32^P]-labeled T7 bacteriophage RNA polymerase transcript, RNA was extracted from each fraction using the procedures described in Additional file 1, and the amount of radiolabeled RNA was determined by Cherenkov counting in a scintillation counter. The results are depicted as the percentage of cpm in each fraction relative to the average cpm in all of the fractions in each gradient. Fraction 1 of the sucrose gradients contains RNA extracted from the pellet.

Quantification of polysomal loading requires recovery of equal amounts of RNA from every fraction, dissolving the RNA in a constant final volume, and analysis of the same proportion of RNA from each fraction. The obstacles to achieving these goals include extraction procedures that are incompatible with density gradients (Additional file 1), differential loss of pellets, differential extraction of RNAs from different regions of gradients, and difficulty in dissolving RNA pellets in Nycodenz gradient fractions containing the peak of ribosomal RNA. With respect to the second problem, sucrose gradient analyses often contain one or more fractions that are devoid of mRNA due to the total loss of a pellet [2,12,31,32]. The partial loss of pellets is more troublesome because it is not immediately obvious. In addition, it is more difficult to extract RNA from fractions at the top of the sucrose gradients, which are usually grossly overloaded with protein, than fractions from the polysomal region, which contain relatively little protein [5]. Additional file 1 describes a reliable protocol using glycogen azure as a co-precipitant that yields equal proportions of RNA from all gradient fractions based on experiments in which each fraction is spiked with a constant amount of [^32^P]-labeled bacteriophage RNA polymerase transcript and the final recovery of radioactivity in each fraction is measured by scintillation counting (Figure 3).

Quantification of polysomal loading requires accurate measurement of mRNA levels which can vary by up to 100-fold in various fractions, such as RT-qPCR, phosphoimage analysis of Northern blots or RNA invader assays [33]. The fact that the linear range of x-ray film is less than a factor of ten makes it difficult to compare the proportion of polysomal mRNA in different gradients by autoradiography, especially if the exposures are not closely matched [34]. Semiquantitative RT-PCR can grossly exaggerate the levels of polysomal mRNA in cell populations in which translation is strongly repressed, because the levels of PCR products in fractions containing high levels of template reach a plateau in fewer cycles than do those in fractions containing low levels of template [34].

Estimating the polysome loading of specific mRNAs is complicated by uncertainties mentioned above. Sucrose gradient analyses of mRNAs that are expressed in meiotic and haploid spermatogenic cells in mice and rats always exhibit a prominent, sharp peak of free-mRNPs near the top of the gradient and a less prominent, broader peak of polysomal mRNA, separated by a valley of one or more fractions containing lower levels of mRNA [1,2,4-9,19-21]. The free-mRNPs of some mRNA species appear to be heterogeneous in size with a peak that sediments slower than monosomes and some free-mRNPs that sediment more rapidly than monosomes [5]. We do not know whether mRNAs in the pellet should be classified as large inactive free-mRNPs or polysomal mRNAs. To estimate polysomal loading, we identify the fractions with the lowest levels of mRNA between the peaks of free-mRNPs and polysomal mRNA, and assign half of the amount of mRNA in that fraction and all of the rapidly sedimenting mRNA including the mRNA in the pellet to the polysomal fraction. We use the same method to divide the mRNAs in Nycodenz gradients into free-mRNP and polysomal mRNAs as illustrated in Figure 1. The validity of this approach is based on agreement of Nycodenz and sucrose gradient results and correlations with translational activity discussed below.

## Conclusions and perspectives

The basic premise of this article is that quantification provides more compelling insights into the mechanism of translational regulation in spermatogenic cells than does non-quantitative analysis. The preceding mentions a series of problems, all of which, unfortunately, are evident in the literature on translational regulation in spermatogenic cells.

All of the gradient analyses from this lab during the past 15 years have been quantified, including >20 mRNA species in adult testis and six mRNAs containing various combinations of the *Smcp *and *Gfp *UTRs in 21 dpp and adult testis [4-6,8]. Our measurements of polysomal loading of the *Ldhc *and *Smcp *mRNAs, summarized in Table 1, have remained consistent despite changes in personnel and techniques of RNA extraction, gradient analysis, and mRNA quantification. Table 1 also indicates that Nycodenz and sucrose gradients and RT-qPCR and phosphoimage analysis of Northern blots and slot blots produce similar estimates of polysomal loading, with the exception of the lower polysomal loading of the *Ldhc *mRNA in 21 dpp and adult testis in Nycodenz gradients compared with sucrose gradients. The biologically important numbers are that ~3.9% of the *Smcp *mRNA (the average of the 11 determinations in Table 1) sediment with polysomes in 21 dpp testis, and 35% of translationally active *Smcp *mRNAs are loaded on polysomes in adult testis.

There is very little quantitative information from other labs to which our measurements can be compared. We note that the 3.9% of *Smcp *in polysomal mRNA in 21 dpp testis is ~2.4-fold less than the estimate that 9.3% of *Prm1 *mRNA is polysomal in 25 dpp testis, a stage when the *Prm1 *mRNA is thought to be translationally inactive [16]. Our estimate for the *Smcp *mRNA in 21 dpp testis is 5- to 10-fold lower than those obtained by RT-qPCR and microarray analysis in 22 dpp testis, 19 and 39%, respectively [3]. We believe those numbers are much too high, and do not accurately reflect the strong repression of the *Smcp *mRNA in early spermatids [4,6,18].

There are also at least two ways in which the present techniques can be improved:

First, increasing the accuracy of measurement of the levels of mRNAs in gradient fractions will reduce the number of replicate gradients required to detect statistical differences. The RNA Invader assay (Third World Technologies, Inc) is reputed to be less variable and nearly as sensitive as RT-qPCR [33].

Second, it is unclear what proportions of the 3.9% of the *Smcp *mRNA that sediment in the polysomal region of sucrose and Nycocdenz gradients in 21 dpp testes are polysomal mRNA and free-mRNP contaminants [4]. The low levels of *Smcp *mRNA in the polysomal regions should not obscure the importance of this question. Premature mRNA translation in spermatids reduces male fertility [35,36], a trait which is under strong evolutionary selection [37]. Thus, strong translational repression mediated by multiple mechanisms in early spermatids may be required to maximize male reproductive success. Perhaps, the levels of polysomal mRNA in repressed cell populations can be accurately measured by combining Nycodenz gradient analysis with immunoprecipitation of polysomes [16].

Finally, translational regulation of individual mRNAs can analyzed by a recently developed technique, ribosome profiling, in which ribosome-protected RNase digestion fragments are deeply sequenced [38]. This technique can be used to obtain estimates of polysome loading as well insights into a variety of regulatory mechanisms reflecting selective associations of ribosomes with specific mRNA sequences.

## Competing interests

The authors declare that they have no competing interests.

## Authors' contributions

KCK conceived of the project, assisted in several experiments, and wrote the manuscript; SKH and JB did most of the experiments, and LC performed the analysis of mRNA translation in purified pachytene spermatocytes. All authors have read and approved the manuscript.

## Supplementary Material

Additional file 1 **Methods for analysis of mRNA translation using sucrose and Nycodenz gradients**. Click here for file

Additional file 2 Figure S1: Equipment for pouring sucrose gradients. Click here for file

Additional file 3 Figure S2: Equipment for collecting fractions from sucrose and Nycodenz gradients. Click here for file

Additional file 4 Figure S3: Changes in absorbance during the analysis of a sucrose gradient. Click here for file

## References

[B1] Kleene KC Patterns of translational regulation in the mammalian testis Mol Reprod Dev 1996 43 268 281 10.1002/(SICI)1098-2795(199602)43:2<268::AID-MRD17>3.0.CO;2-# 8824926

[B2] Schmidt EE Hanson ES Capecchi MR Sequence-independent assembly of spermatid mRNAs into messenger ribonucleoprotein particles Mol Cell Biol 1999 19 3904 3915 10207114 10.1128/mcb.19.5.3904 PMC84248

[B3] Iguchi N Tobias JW Hecht NB Expression profiling reveals meiotic male germ cell mRNAs that are translationally up- and down-regulated Proc Natl Acad Sci USA 2006 103 7712 7717 10.1073/pnas.0510999103 16682651 PMC1472510

[B4] Bagarova J Chowdhury T Kimura M Kleene KC Identification of elements in the Smcp 5' and 3' UTR that repress translation and promote the formation of heavy inactive mRNPs in spermatids by analysis of mutations in transgenic mice Reproduction 1010 140 853 864 10.1530/REP-10-0323 20876225

[B5] Cataldo L Mastrangelo MA Kleene KC A quantitative sucrose gradient analysis of the translational activity of 18 mRNA species in testes from adult mice Mol Hum Reprod 1999 5 206 213 10.1093/molehr/5.3.206 10333353

[B6] Hawthorne SK Busanelli RR Kleene KC The 5'UTR and 3'UTR of the sperm mitochondria-associated cysteine-rich protein mRNA regulate translation in spermatids by multiple mechanisms in transgenic mice Dev Biol 2006 297 118 126 10.1016/j.ydbio.2006.04.468 16759650

[B7] Kleene KC PolyA, shortening accompanies the activation of translation of five mRNAs during spermiogenesis in the mouse Development 1989 106 367 373 2512111 10.1242/dev.106.2.367

[B8] Kleene KC Cataldo L Mastrangelo MA Tagne JB Alternative patterns of transcription and translation of the ribosomal protein L32 mRNA in somatic and spermatogenic cells in mice Exp Cell Res 2003 291 101 110 10.1016/S0014-4827(03)00339-2 14597412

[B9] Kleene KC Multiple controls over the efficiency of translation of the mRNAs encoding transition proteins, protamines, and the mitochondrial capsule selenoprotein in late spermatids in mice Dev Biol 1993 159 720 731 10.1006/dbio.1993.1277 8405691

[B10] Mathews MB Sonenberg N Hershey JWB Sonenberg N, Hershey JWB, Mathews MB Origins and principles of transational control Translational Control of Gene Expression 2000 Cold Spring Harbor, NY: Cold Spring Harbor Laboratory Press 1 32

[B11] Arava Y Wang Y Storey JD Liu CL Brown PO Herschlag D Genome-wide analysis of mRNA translation profiles in Saccharomyces cerevisiae Proc Natl Acad Sci USA 2003 100 3889 3894 10.1073/pnas.0635171100 12660367 PMC153018

[B12] Herbert TP Hecht NB The mouse Y-box protein, MSY2, is associated with a kinase on non-polysomal mouse testicular mRNAs Nucleic Acids Res 1999 27 1747 1753 10.1093/nar/27.7.1747 10076007 PMC148379

[B13] Tafuri SR Familari M Wolffe AP A mouse Y box protein, MSY1, is associated with paternal mRNA in spermatocytes J Biol Chem 1993 268 12213 12220 8505341

[B14] Houssais JF Rickwood D Fractionation of ribonucleoproteins from eukaryotes and prokaryotes Iodinated Density Gradient Media 1983 Washington, DC: IRL Press 43 66

[B15] Antic D Keene JD Messenger ribonucleoprotein complexes containing human ELAV proteins: interactions with cytoskeleton and translational apparatus J Cell Sci 1998 111 183 197 9405302 10.1242/jcs.111.2.183

[B16] Sanz E Yang L Su T Morris DR McKnight GS Amieux PS Cell-type-specific isolation of ribosome-associated mRNA from complex tissues Proc Natl Acad Sci USA 2009 106 1 3939 13944 10.1073/pnas.0900879106 PMC2728999 19666516

[B17] Ruan H Shantz LM Pegg AE Morris DR The upstream open reading frame of the mRNA encoding S-adenosylmethionine decarboxylase is a polyamine-responsive translational control element J Biol Chem 1996 271 29576 29582 10.1074/jbc.271.12.6789 8939886

[B18] Cataldo L Baig K Oko R Mastrangelo MA Kleene KC Developmental expression, intracellular localization, and selenium content of the cysteine-rich protein associated with the mitochondrial capsules of mouse sperm Mol Reprod Dev 1996 45 320 31 10.1002/(SICI)1098-2795(199611)45:3<320::AID-MRD9>3.0.CO;2-U 8916043

[B19] Alcivar AA Trasler JM Hake LE Salehi-Ashtiani K Goldberg E Hecht NB DNA methylation and expression of the genes coding for lactate dehydrogenases A and C during rodent spermatogenesis Biol Reprod 1991 44 527 535 10.1095/biolreprod44.3.527 2015369

[B20] Fujimoto H Erickson RP Toné S Changes in polyadenylation of lactate dehydrogenase-X mRNA during spermatogenesis in mice Mol Reprod Dev 1988 1 27 34 10.1002/mrd.1080010106 2908441

[B21] Persengiev SP Raval PJ Rabinovitch S Millette CF Kilpatrick DL Transcription factor Sp1 is expressed by three different developmentally regulated messenger ribonucleic acids in mouse spermatogenic cells Endocrinology 1996 137 638 646 10.1210/en.137.2.638 8593813

[B22] Thermann R Hentze MW Drosophila miR2 induces pseudo-polysomes and inhibits translation initiation Nature 2007 447 875 878 10.1038/nature05878 17507927

[B23] Wang B Yanez A Novina CD MicroRNA-repressed mRNAs contain 40S but not 60S components Proc Natl Acad Sci USA 2008 105 5343 5348 10.1073/pnas.0801102105 18390669 PMC2291078

[B24] Eulalio A Huntzinger E Izaurralde E Getting to the root of miRNA-mediated gene silencing Cell 2008 132 9 14 10.1016/j.cell.2007.12.024 18191211

[B25] Arava Y Boas FE Brown PO Herschlag D Dissecting eukaryotic translation and its control by ribosome density mapping Nucleic Acids Res 2005 33 2421 2432 10.1093/nar/gki331 15860778 PMC1087779

[B26] Steensgaard J Humphries S Spragg SP Rickwood D, Hames BD Measurements of sedimentation coefficients Preparative Centrifugation. A Practical Approach 1992 Washington, DC: IRL Press 187 232

[B27] Fajardo MA Haugen HS Clegg CH Braun RE Separate elements in the 3' untranslated region of the mouse protamine 1 mRNA regulate translational repression and activation during murine spermatogenesis Dev Biol 1997 91 42 52 10.1006/dbio.1997.8705 9356170

[B28] Schmidt EE Schibler U Developmental testis-specific regulation of mRNA levels and mRNA translational efficiencies for TATA-binding protein mRNA isoforms Dev Bio 1997 184 138 149 10.1006/dbio.1997.8514 9142990

[B29] Lolicato F Marino R Paronetto MP Pellegrini M Dolci S Geremia R Grimaldi P Potential role of Nanos3 in maintaining th undifferentiated spermatogonia population Dev Biol 2008 313 725 738 10.1016/j.ydbio.2007.11.011 18089289

[B30] Bellvé AR Cavicchia JC Millette CF O'Brien DA Bhatnagar YM Dym M Spermatogenic cells of the prepuberal mouse. Isolation and morphological characterization J Cell Biol 1977 74 68 85 874003 10.1083/jcb.74.1.68 PMC2109873

[B31] Agrawal MG Bowman LH Transcriptional and translational regulation of ribosomal protein formation during mouse myoblast differentiation J Biol Chem 1987 262 4868 4875 3558374

[B32] Petersen CP Bordeleau ME Pelletier J Sharp PA Short RNAs repress translation after initiation in mammalian cells Mol Cell 2006 21 533 542 10.1016/j.molcel.2006.01.031 16483934

[B33] Murray EL Schoenberg DR Application of the Invader RNA assay to the polarity of vertebrate mRNA decay Methods Mol Biol 2008 419 259 276 full_text 18369989 10.1007/978-1-59745-033-1_18 PMC2715152

[B34] Sambrook J Russell DW Molecular Cloning, A Laboratory Manual 2001 Cold Spring Harbor, NY: Cold Spring Harbor Press

[B35] Lee K Haugen HS Clegg CH Braun RE Premature translation of protamine 1 mRNA causes precocious nuclear condensation and arrests spermatid differentiation in mice Proc Natl Acad Sci USA 1995 92 12451 12455 10.1073/pnas.92.26.12451 8618919 PMC40375

[B36] Tseden K Topaloglu O Bohm D Wolf S Muller C Adham I Meinhardt A Dev A Schluter G Engel W Nayernia K Premature translation of transition protein 2 mRNA causes sperm abnormalities and male infertility Mol Reprod Dev 2007 74 273 279 10.1002/mrd.20570 16967499

[B37] Kleene KC Sexual selection, genetic conflict, selfish genes and the atypical patterns of gene expression in spermatogenic cells Dev Biol 2005 277 16 26 10.1016/j.ydbio.2004.09.031 15572136

[B38] Ignolia NT Ghaemmaghami S Newman JRS Weissman JS Genome-wide analysis in vivo of translation with nucleotide resolution using ribosome profiling Science 2009 324 218 223 10.1126/science.1168978 19213877 PMC2746483

