# Supplementary material for: Quantitative analysis of mRNA translation in mammalian spermatogenic cells with sucrose and Nycodenz gradients
Source: Reprod Biol Endocrinol. 2010 Dec 25;8:155. doi: 10.1186/1477-7827-8-155 (PMC3022843; doi:10.1186/1477-7827-8-155)
Supplement: Additional file 1 — Methods for analysis of mRNA translation using sucrose and Nycodenz gradients. [file 1477-7827-8-155-S1.DOC]

# **Additional file 1. Methods for analysis of mRNA translation using sucrose and Nycodenz gradients.**

There are many descriptions of methods of preparing and using sucrose gradients to analyze mRNA translation. Some of these articles can be identified with Google but not with Entrez. Several articles are highly recommended because they contain valuable discussions of technical details and using sucrose gradients to elucidate mechanisms of translational regulation [1-3].

The protocols described below use the Beckman SW60Ti rotor, which features the smallest tubes of any swinging bucket rotor. This rotor offers several advantages in analyzing mRNA translation in spermatogenic cells, the most important being that the small tubes keep the concentration of ribosomes high, which makes it possible to obtain absorbance tracings of extracts from the testes of one 21 dpp prepubertal mouse and purified spermatogenic cells. In addition, the small tubes produce ~10 ~0.4 ml sucrose gradient fractions, a convenient size for RNA extraction in 1.5 ml tubes. Although rotors with longer buckets, such as the SW40Ti, provide greater resolution, the resolution of the SW60Ti is sufficient in our experiments because we analyze small mRNAs with short coding regions that sediment in a region of the gradient in which the peaks of polysomes containing known numbers of ribosomes can be resolved in absorbance tracings. Our estimates of polysome size and ribosome spacing are virtually identical using the SW60Ti and SW40Ti rotors [4-6]. We always extract the RNA from the pellets on the bottom of the ultracentrifuge tubes of sucrose gradients which contain significant amounts of certain mRNAs [4-6]. The SW60Ti rotor is also suitable for Nycodenz gradients because the tubes are similar in size to those of the Beckman SW50 rotor which is used in other labs [7,8].

Note: *Separation of free-mRNPs and polysomes by ultracentrifugation of sucrose and Nycodenz gradients is a potentially dangerous procedure which requires careful attention to details to avoid accidents such as that as described* in [**www.ehs.cornell.edu/chem_lab_safety/centrifuge_safe.cfm**](http://www.ehs.cornell.edu/chem_lab_safety/centrifuge_safe.cfm)**.** ***A complete description of the use and care of ultracentifuges, rotors and tubes is beyond the scope of the*** *present article, and the reader is referred to the on-line Care Guide for Sorvall Rotors*, **www.axeb.dk/pdfs/application_briefs/Sorvall/6.../s00039.pdf,**

*and the Beckman Coulter Rotor Safety Guide*, [www.beckmancoulter.com/resourcecenter/labresources/.../rotor.pdf](http://www.beckmancoulter.com/resourcecenter/labresources/.../rotor.pdf). *Besides the ultracentrifuges in the authors’ laboratory may not be available in other universities.*

*General lab procedures*

*Materials*

1. Gradient former (15 ml, Hoefer Scientific Cat. No. SG15) should be suitable for pouring small sucrose gradients for the SW60Ti and SW40Ti rotors. An 18 gauge 1 inch syringe needle with the sharp tip broken off is attached to the nipple on the gradient former. The front and back back chambers are labeled as follows: front chamber, 40% sucrose and a line marking the level of 1.83 ml; back chamber, 15% sucrose and a line marking the level of 2.11 ml. Stir bars cut out of Teflon triangles (Fisher Scientific, Cat. No. 1451373) are short enough to fit the chambers and fat enough to stir effectively. The gradient former that we actually use for SW60Ti gradients has a capacity of 10 ml, was purchased from Hoefer Scientific, and is not commercially available.

2. Polyallomer centrifuge tubes for the Beckman SW60Ti rotor (Beckman Coulter, Cat. No. 328874).

3. 50 µl glass capillary pipettes (VWR Cat. No. 53432-783), baked 3 hrs at 250^o^C in a glass test tube with a metal cap.

4. 5-3/4 inch Pasteur pipettes, baked 3 hrs at 250^o^C in a 1 L beaker covered with aluminum foil.

5. A peristaltic pump capable delivering a flow rate between 0.5 and 10 ml/min, such as Cole-Parmer Masterflex L/S Compact, Variable-Speed Pump R-07554-80 equipped with the L/S13 R-07013-20 standard pump head and L/S13 06424-13 C-flex tubing.

6. Glass Teflon homogenizer: Wheaton tapered glass tissue grinder, 1 ml (VWR Cat no. 12431-524). The top of the homogenizer tube is covered with aluminum foil and baked. Add 10 µl DEPC to 10 ml DH_2_O in a 14 ml polypropylene, cap and shake vigorously, add the Teflon pestle and incubate overnight at 25^o^C, then wrap the Teflon- pestle end in aluminum foil leaving ~3 cm of metal shaft exposed, and autoclave 20 min.

7. Magnetic stirrer (Cole-Parmer, MicroV, Cat. No. 4805). This stirrer has been discontinued but is sold as a used item.

8. Beckman Altex model 153 UV-detector with 2 mm semipreparative flow cell.

9. Motor driven homogenizer (Wheaton Overhead Stirrer, VWR Cat. No. 62400-904).

*Solutions*

1. We use two kinds of diethyl pyrocarbonate-treated DH_2_O. Freshly-prepared DEPC-DH_2_O, "FPD-DH_2_O", is prepared by adding 100 µl DEPC (Sigma-Aldrich Cat. No. D5758, stored at -80^o^C) to 100 ml DH_2_O in a baked 250 ml Erlenmeyer flask. The flask is covered with Parafilm, the mixture is shaken vigorously and the FPD-DH_2_O is used immediately to inactivate RNase in the gradient former and tubing for pouring and collecting gradients.

To prepare autoclaved DEPC-DH_2_O, "AD-DH_2_O", FPD-DH_2_O is incubated at 25^o^C overnight and autoclaved 20 min to degrade the DEPC.

Note: *DEPC is a known carcinogen: wear gloves and work in a fume hood.*

2. HNM buffer is used in the preparation of the cytoplasmic extracts and sucrose and Nycodenz gradients: 100 mM NaCl, 3 mM MgCl_2_, 20 mM HEPES, pH 7.6. HEPES contains amines which inactivate DEPC and is degraded by autoclaving. Dissolve 5.85 g NaCl and 0.609 g MgCl_2·_6H20 in 1 l DH_2_O, add a DEPC-treated magnetic stir bar and 1.0 ml DEPC, cover with Parafilm, shake vigorously and incubate over night at 25^o^C. Replace the Parafilm with aluminum foil, autoclave 20 min, cool to 25^o^C, add 4.7 g HEPES free-acid and adjust the pH to 7.6 with 10 N NaOH. Measure the pH of the HNM by pouring small aliquots into a tube without allowing the main stock to contact the pH meter electrode.

3. 15 and 40% sucrose (w/w) in HNM buffer are prepared by dissolving respectively 15 and 40 g of RNase-free sucrose (Sigma-Aldrich Cat. No. S0389) in 85 and 60 ml of HNM buffer. Sucrose gradient solutions are stored at -20^o^C. Thaw sucrose gradient solutions and swirl until the absence of Schlieren demonstrates that the solution is homogenous.

4. 10% Triton X-100 (Sigma-Aldrich, Cat. No. T-8787) (v/v) is prepared in AD-DH_2_O, and stored at -20^o^C

5. Glycogen azure (Sigma-Aldrich, Cat. No. G5510), rabbit liver glycogen covalently linked to Remazol Brilliant Blue R, is a useful co-precipitant which facilitates visualizing alchohol pellets containing small amounts of DNA and RNA. Glycogen azure is dissolved at 5 mg/ml in HNM buffer containing 0.5% SDS, digested with proteinase K (100 mg/ml) for two hours at 50^o^C, extracted twice with phenol:chloroform (1:1), and precipitated with two volumes of ethanol. The precipitate is collected by centrifugation at 10,000 X G for 10 min, the pellet is washed twice with 100% ethanol, air dried and redissolved at the orginal volume in AD-DH_2_O. The stock is stable for at least two years at -20^o^C.

6. Phenol:chloroform:isoamyl alcohol (25:24:1) (Sigma-Aldrich Cat. No. P2069).

7. DEPC-treated 5 M NaCl. Dissolve 14.6 g NaCl in sufficient DH_2_O to make a total volume of 50 ml. Add 50 μl DEPC, shake vigorously, incubate at room temperature overnight, autoclave 20 min and store at -20^o^C.

8. DEPC-treated 75% ethanol. Combine 37.5 ml 100% ethanol, 12.5 ml DH_2_0 and 50 μl DEPC and vortex. Incubate overnight at 25^o^C, and store at -20^o^C.

*Pouring sucrose gradients*

1. Cut a 60 cm piece of C-flex tubing (Cole Parmer L/S13 06424-13) with a clean razor

blade (VWR 55411-050). Assemble the tubing on the gradient former and the peristaltic pump and add the stir bar to the front chamber of the gradient former. Place the gradient former on the magnetic stirrer. The equipment for pouring a sucrose gradient is depicted in Additional file 2, Figure S1.

1. Open the front and back stopcocks on the gradient former (handles pointing

parallel to bench top). Fill both chambers of the gradient former with FPD-DH_2_O. Run the pump at ~0.5 ml/min until both chambers are dry. Repeat several times over a period of 30-60 min.

1. Run the pump until the FPD-DH_2_O is absent from the gradient maker chambers

and ceases to flow from the capillary pipette. Close the stopcocks on the gradient former, both handles pointing up.

1. Using a baked Pasteur pipette, add 2 ml 15% sucrose to the back chamber of the

gradient former. Open the back stopcock slowly and carefully until the 15% sucrose begins to flow into the front chamber; then immediately close the back stopcock. Now, open the front stopcock and operate the pump until the front chamber is empty and liquid ceases flowing from the capillary pipette. This procedure eliminates a bubble which often forms in the channel connecting the front and back chambers.

1. With pump off both and both stopcocks closed, fill the front and back chambers with

40% and 15% sucrose respectively to the level of the lines. Note: The volume of 15% sucrose in the back chamber, 2.11 ml, is slightly greater than that of the 40% sucrose in the front chamber, 1.83 ml, to ensure that 40% sucrose does not flow into the back chamber when the channel connecting the chambers is opened.

1. Place a polyallomer centrifuge tube (Beckman Coulter, Cat. No 328874) in a clear

plastic rack, and place the capillary pipette in the centrifuge tube.

1. Turn on the magnetic stirrer to the highest setting. Open both stopcocks, and turn

on the peristaltic pump at a flow rate of ~1 ml/min. The gradient is poured manually. Start with the capillary tube just above the bottom of the centrifuge tube which is tilted slightly. As the gradient fills the tube, gradually raise the pipette tip always keeping it several mm above the meniscus, allowing the gradient to flow down the side of the tube onto the top of the gradient.

Note: *Watch the gradient former carefully while pouring the gradient to avoid two mishaps. First, the stir bar gets stuck and ceases mixing the solutions in the front chamber. Second, a bubble lodges in the narrow channel connecting the front and back chambers, temporarily preventing the flow of 15% sucrose into the front chamber. Both mishaps produce a gradient with a sharp interface between sucrose solutions of high and low concentration which is visble as an artifactual peak in the absorbance tracing.*

1. Pour additional gradients starting with step 4 above.
2. After all of the gradients have been poured, weigh the tubes. Take the lightest

gradient and remove the top until the meniscus is ~5 mm from top of the tube using a P200 and a yellow tip. Now weigh the tube again. Then remove solution from the top of the remaining gradients with a yellow tip and a P200 until all of the gradients have the same weight with an accuracy of ≤30 mg. The “Drosophila anesthetizer” (Carolina Science, Cat. No.DH-173040) provides a convenient holder for weighing SW60Ti tubes.

Note: *Ultracentrifuge tubes must be balanced with gradients of the same composition. Balancing tubes by weight only is potentially dangerous, because the centrifugal force on each tube is determined by both the density and radius. The implications of this line of reasoning is illustrated by considering that gradients which differ in weight by 30 mg at 1 x g, differ in weight by 4.2 kg at 140,000 x g.*

1. To chill the gradients, cover the tops of the ultracentrifuge tubes with Parafilm. The

gradients can be poured the day before the experiment and chilled overnight in a cold room, or the gradients can be chilled in a -20^o^C freezer for ~15 min and placed in a cold room for one hour.

11. After pouring the gradients, wash the gradient former using FPD-DH_2_O warmed to ~50^o^C in a microwave. (At ~50^o^C, the flask will be hot to the touch but not so hot as to be painful.) Fill both chambers with FPD-DH_2_O and run the pump until the chambers are dry. Repeat at least five times. If sucrose dries in the gradient former, the channel and outlet needle can become clogged and a stopcock may be frozen. Applying excessive pressure to the stopcock can break the gradient former.

*Getting ready to prepare the cytoplasmic extract and sediment sucrose gradients*

Before euthanizing the mouse, assemble all of the materials listed below, place them in the correct place (indicated by brackets), and chill as needed. As a matter of good technique, minimize the interval between sacrificing the mouse and preparing extract. In addition, everything that comes in contact with the testes and extract must be cold. It is important to work rapidly as soon as the mouse is sacrificed because ribosomes begin to run off the mRNA, which could cause erroneously low estimates of polysome loading. However, in several experiments we euthanized a mouse, and prepared one testis as a cytoplasmic extract immediately, and allowed the other testis to sit at room temperature on Parafilm for five minutes before preparing the extract. The absorbance tracings of these gradients were virtually identical implying that there is little polysome breakdown in five minutes.

1. Turn on the ultracentrifuge, set the temperature to 4^o^C, and turn on the vacuum. The vacuum pump should begin to work and the temperature should begin to decrease immediately. When the chamber has cooled to 4^o^C, carefully transfer the rotor from the cold room to the ultracentrifuge and place it on the drive shaft. The ultracentrifuge rotor and buckets are precooled overnight in a cold room.

2. 0.5% Triton X100 in HNM buffer (add 50 µl 10% Triton X100 to 950 µl HNM buffer) {ice bucket in cold room}.

3. Baked Pasteur pipettes {cold room}

4. Rubber bulbs for Pasteur pipettes (VWR, Cat. No. 56311-060) {cold room}

5. 1 ml baked glass homogenizer tube {ice bucket in cold room}

6. Insert the shaft of the Teflon pestle into the chuck of the motor driven homogenizer in the {cold room} and tighten with the key until the shaft of the shaft is firmly held by the chuck. To avoid touching the bottom of the metal shaft or the Teflon tip with your glove, hold the shaft in the aluminum foil in which it was autoclaved.

7. Two small ice buckets, one in the cold room and one at room temperature where the mouse will be euthanized and dissected.

8. Label a series of 1.5 ml tubes with numbers, one series for the cytoplasmic extracts {ice bucket in cold room} and the other for tail clips (if needed) {dissection area}. The numbers will correspond to the buckets in the ultracentrifuge rotor that will contain each extract.

9. Place a cold-room compatible micro-centrifuge (such as VWR Galaxy 14D,

Catalogue No. 37001-296) in the {cold room} overnight. Eppendorf microcentrifuges are not cold-room compatible.

10. 1000 µl and 20 µl Pipettemen and autoclaved blue and yellow tips {cold room}. Set the P20 to 8 µl and the P1000 to 320 µl.

11. Place RNasin ribonuclease inhibitor (Promega-Biotec N2511) in the {ice bucket in cold room}.

12. While the above items are cooling, get the following items ready for the dissection: forceps, small scizzors (Miltex 5-304, VWR, Cat. No. 21909-404), 75% ethanol in a squirt bottle, a plastic bag for the carcasses, Parafilm for dissecting the testes, paper towels, and Kimwipes.

13. Fetch male mice from the animal room. Plan on sedimenting one testis from an adult male or both testes from one 21 dpp prepubertal male on each SW60Ti gradient.

*Preparing the Cytoplasmic Extract and Starting the Ultracentrifuge*

Before euthanizing a mouse, make sure that every item listed above has been placed in the right place and has been chilled appropriately, including the ultracentrifuge chamber, rotor and buckets.

1. Place the glass homogenizer tube in an ice bucket and bring to the dissection area. Euthanize the mouse by CO_2_ hypoxia, squirt 75% ethanol on the abdomen, wipe off the excess ethanol with a Kimwipe, dissect out testes and transfer to Parafilm, remove the *tunica albuginea,* transfer the mass of seminiferous tubules to the homogenizer tube in the ice bucket, shake the tubules to the bottom of the homogenizer tube, insert the homogenizer tube into the ice bucket, and go immediately to the cold room.

2. Add 320 µl HNM with 0.5% Triton X100 to the tubules in the homogenizer tube, and lyse the testes with 10 up-and-down strokes with a motor-driven homogenizer set at position 5. Hold the homogenizer tube securely, watch homogenizer tube carefully on the upstroke, and adjust the speed of the upstroke so that the homogenate does not swirl out of the tube.

3. Turn off the homogenizer motor, transfer the lysate with a chilled, baked Pasteur pipette to a 1.5 ml tube, and place the tube in an ice bucket.

4. Using AD-DH_2_O and a chilled, baked Pasteur pipette wash off the pestle and the homogenizer tube, and shake the last drops of AD-DH_2_O out of the homogenizer tube.

5. Repeat steps 1-4 until all of the testes have been homogenized.

6. Add 8 µl RNasin to each cytoplasmic lysate, vortex several seconds to mix, and centrifuge top speed (~13,000 x G) for 2 min in the microcentrifuge.

7. Using a P1000, carefully layer 250 µl of the cytoplasmic extract on top of the sucrose gradient, being careful not to transfer any nuclear pellet. To minimize mixing while adding the cytoplasmic extract, use a P1000 that operates smoothly and can be precisely controlled. Keep the P1000 nearly parallel to the meniscus of the gradient with the tip of the blue tip touching the wall of the centrifuge tube just above the top of the gradient and expel the lysate slowly. After layering 250 µl of cytoplasmic lysate on the top of the gradient, the tube should be filled to ~2 mm of the top.

Place the centrifuge tubes in the ultracentrifuge buckets and screw the lids on tightly, so the bucket is sealed to eliminate evaporation during the centrifuge run. If necessary, add 250 µl of buffer to an extra gradient to prepare a balance tube. It should not be necessary to weigh the tubes again if the weights of the tubes were adjusted carefully after pouring the gradients and the same amount of lysis buffer was layered on each gradient.

8. Release the vacuum on the ultracentrifuge and open the cover. Remove the rotor from the drive shaft, and place it on the strand. Place all of the buckets in the slots on the rotor, being careful to match the numbers on the buckets with the positions on the rotor.

9. Place the rotor with buckets on the drive shaft, close the cover, turn on the vacuum.

10. Program the ultracentrifuge: 35,000 rpm; 4^o^C; 80 min; acceleration, 1; deceleration,

7, and sart the run.

*Prepare to collect gradient fractions and obtain absorbance tracings*

During the ultracentrifuge run, get ready to collect the gradient fractions.

1. Label a sufficient number of autoclaved 1.5 ml tubes to collect the fractions from each gradient: ~11 tubes plus a tube for the pellet of each sucrose gradient.

2. Add 0.32 g guanidine thiocyanate (Promega Cat. No. V2791) to each tube that will contain gradient fractions. It is much easier to weigh small amounts of granular than lumpy guanidine thiocyanate. The 1.5 ml tube that will contain the pellet on the bottom of the ultracentrifuge tube does not contain guanidine. Centrifuge the tubes for 1 min at 13,000 X G to pack the guanidine thiocyanate. Add 0.4 ml DH_2_O to an extra tube containing 0.32 g guanidine thiocyanate, and draw a line at the meniscus without dissolving the guanidine thiocyante. Then draw the line at the same position on all of the tubes. Wear a particle mask while weighing guanidine thiocyanate; this chemical is intensely irritating to mucus membranes.

3. Prepare 100 ml of FPD-DH_2_O as described above. Have available baked full length 50 µl capillary pipettes and baked, broken 50 µl capillary pipettes about 3 cm long. To break capillary pipettes, score with a diamond point and snap, place in a glass scintillation vial, cover with aluminum foil and bake. Insert the full-length capillary pipette into the FPD-DH_2_O and the 3 cm segment of capillary tubing into the mouth of a baked 125 ml Erlenmeyer flask. Operate the pump at low speed for one hour.

4. When the centrifuge run is finished, release the vacuum, open the chamber, transfer the rotor to the stand, close the chamber, remove the buckets and place them in the bucket holder, and immediately transfer the buckets to the cold room.

*Absorbance tracings of sucrose gradients*

Sucrose gradients are collected from the top and from the bottom. It is generally recognized that collecting gradients from the top produces better resolution than collecting from the bottom [3]. We have collected gradients by both methods, and agree with this assessment as it applies to absorbance tracings. However, our lab normally analyzes the distribution of mRNAs in 10 fractions from sucrose gradients, and the resolution of gradients collected from the top and bottom is similar. Currently, we collect all of our gradients from the bottom because this method is less prone to accidents, an important consideration in working with hard-to-obtain cell samples such as purified spermatogenic cells and staged prepubertal transgenic mice. Additional file 3, Figure S2, panel A, depicts the configuration of the equipment to obtain an absorbance tracing and collect fractions from a sucrose gradient.

We prefer to collect fractions manually because this produces fractions of more uniform size than those obtained with drop counter and a fraction collector. The size of the drops of density gradients increases progressively from bottom to top in accordance with decreasing density, but the drop-size decreases at the top resulting from the decreased surface tension of the non-ionic detergent in the lysis buffer.

The paucity of absorbance tracings of sucrose gradients of testis and purified spermatogenic cells in the literature probably has two causes: lack of detailed descriptions of how to obtain absorbance tracings and misconceptions that the amount of ribosomes from purified spermatogenic cells is too small to obtain absorbance tracings. The solution to the second problem is extremely simple: keep the concentration of ribosomes high by using a rotor with small tubes such as the Beckman SW60Ti.

Before describing how to obtain absorbance profiles, it is relevant to note that the absorbance at 254 nm of a sucrose gradient is influenced by several factors in addition to the absorbance of ribosomal RNAs. The UV absorbance of ribosomes is augmented by light scattering, while the absorbance at the top of the gradient represents a combination of small molecules in the extract (nucleotides and tRNAs) and the non-ionic detergent in the lysis buffer. In addition, the quality of the tracing can be degraded by mixing sucrose solutions of different density (Schlieren), or by bubbles in the flow cell of the UV analyzer. The passage of an air bubble through the flow cell produces a transitory increase in absorbance, while the movement of a bubble that is lodged in the flow cell produces a wide zig-zag line.

The resolution of absorbance tracings is degraded by Schlieren caused by mixing two solutions with differing refractive index, for example by collecting a gradient with a capillary tube filled with buffer without 40% sucrose or from the bottom of a gradient which contains a 60% sucrose pad. In other words, Schlieren is minimized by starting to collect the gradient with a capillary tube that is filled with the same sucrose gradient solution as that at the bottom of the gradient.

Calibrate the pump and chart recorder speed for each size of ultracentifuge tube to produce an absorbance tracing of the desired length. Also, keep records of the observed full scale absorbance of every gradient, in relation to the source of the extract (numbers of purified cells, number of testes and age of mouse) and ultracentrifuge tube size. Use this information to predict the full scale absorbance of the UV analyzer. It is convenient to compile this information on a label on the chart recorder.

*Aligning absorbance tracings and fractions*

Estimating the size of polysomes and free-mRNPs of specific mRNAs in Northern blots with absorbance tracings of sucrose gradients requires aligning the fractions and absorbance tracing, because a specific section of the gradient flows through a shorter piece of tubing before it enters the flow cell and a longer piece of tubing before it is collected as a fraction. This can be easily calibrated by pumping H_2_O through the capillary tube to obtain a stable absorbance baseline with the chart recorder on. Then withdraw the capillary tube to suck air and mark the position of three events on the chart: (1) the point at which the capillary tube is removed; (2) the point at which the absorbance increases when air enters the flow cell, (3) the point at which FPD-DH_2_O ceases to flow into the collection tube. Additional file 4 Figure S3, illustrates the use of this information in aligning absorbance tracings and fractions.

*Collecting fractions from a sucrose gradient with absorbance tracing.*

1. About one hour before beginning to collect fractions, assemble the equipment to collect fractions and obtain an absorbance tracing as depicted in Additional file 3, Figure S2, Panel A. Turn on the UV analyzer and pump FPD-DH_2_O slowly through the flow cell during this period.

2. To collect fractions from the bottom of ultracentrifuge tubes, collect several ml of each of the following solutions: 0.1% SDS in AD-DH_2_O, FPD-DH*_2_*O, and 40% sucrose in HNM. To avoid introducing an air bubble into the flow cell, turn the pump off and wait several seconds before transferring the capillary tube from one solution to the next. The entry of the 40% sucrose into the flow cell is marked by an increase in absorbance caused by Schlieren followed by a decrease to baseline (Additional file 4, Figure S3). If there is an air bubble in the flow cell, repeat the washes with 0.1% SDS, AD-DH_2_O and 40% sucrose. Increasing the speed of the pump and the low surface tension of the 0.1% SDS facilitate removing bubbles from the flow cell.

3. If there is no sign of an air bubble, you are ready to begin. Turn the pump off, and open the lids of all of the pre-numbered 1.5 ml tubes to collect the fractions. Insert the capillary tube into the ultracentrifuge tube until it rests on the bottom, being careful not to stir the gradient. Turn the pump on and hit the event marker. When the chart has moved the distance calibrated above, hit the event marker again and begin collecting gradient fractions into each tube until the meniscus is even with the line. Hit the event marker and quickly transfer the short capillary pipette to the next 1.5 ml tube and repeat.

4. When all the fractions have been collected, place the tubes in a shaker in the cold room and mix until the guanidine thiocyanate is dissolved.

5. Dissolve the small transparent pellets on the bottom of the ultracentrifuge tubes in 0.5 ml 4 M guanidine thiocyanate in HNM by pipetting up and down with a baked Pasteur pipette, and transfer to the prelabeled 1.5 ml tube.

6. The gradient fractions can be stored at -80^o^C for months or years before extraction and analysis. Alternatively, the RNAs/RNPs can be precipitated by addition of 5 µl glycogen azure co-precipitant, 65 µl 5 M NaCl and 700 µl isopropanol to each tube, vortex and incubate at -20^o^C.

*Nycodenz gradients*

Since Nycodenz gradients have rarely been used to analyze mRNA translation, articles that use this technique are cited at the end of this section in addition to the references mentioned previously [5,7,8, 9-11].

1. Nycodenz can be purchased from Accurate Chemical and Scientific Corp (Cat. No. AN7050). Nycodenz gradients are prepared from 20%, 30%, 40%, 50% and 60% (w/v) in HNM buffer in conical, screw cap 50 ml tubes. The appropriate amount of Nycodenz is weighed and dissolved in HNM to make a final volume of 20 ml. Since partially hydrated Nycodenz forms sticky masses that are difficult to dissolve, small aliquots of Nycodenz are progressively dissolved in 8 ml HNM by heating at 60^o^C and vortexing. After the Nycodenz is dissolved completely, HNM is added to a final volume of 20 ml according to the lines on the centrifuge tube.

2. Step gradients are prepared by pipetting 760 µl of each Nycodenz solution into polyallomer centrifuge tubes for the SW60Ti rotor, filling the tube to ~5 mm of the top. Pipette carefully, weigh the tubes after each addition and adjust the weights to within 30 mg if necessary. Cover the tubes with Parafilm and pre-cool to 4^o^C before euthanizing the mouse.

3. Prepare cytoplasmic extracts as described above and layer 250 μl on the Nycodenz gradients which are centrifuged at 37,000 rpm for 24-26 hrs at 4^o^C.

4. The tubes to collect Nycodenz gradient fractions do not contain guanidine, but should be labeled with a line to mark the appropriate volume. A line drawn at the point at which a 1.5 ml tube fits into a blue tip will produce ~22 ~0.18 ml fractions per gradient, but we routinely label 24 tubes for each gradient to be safe.

5. The equipment to collect Nycodenz gradients involves the pump, 60 cm piece of tubing, long and short capillary pipettes as depicted in Additional file 3, Figure S2, Panel B. Before collecting fractions from Nycodenz gradients, FPD-DH_2_O is pumped through the tubing for about an hour. The FPD-DH_2_O is pumped out of the tubing, the pump is turned off, and the long capillary pipette is inserted to the bottom of ultracentrifuge tube.

6. Nycodenz gradients fractions are collected as described for sucrose gradients with the pump set at about 1.0 ml/min without the complications of absorbance tracings.

7. When finished collecting the fractions, add 400 μl 5.8 M guanidine thiocyanate in DEPC H_2_O to each fraction, close the lid and vortex. The 5.8 M guanidine thiocyanate is kept in a 37^o^C water bath because it precipitates in the cold room. After mixing, the fractions can be placed at -80^o^C for long term storage, or the RNA/RNPs can be precipitated by addition of 5 µl glycogen azure (5 µg/µl), 50 µl 5 M NaCl, and 590 µl isopropanol to each fraction and incubation at -20^o^C overnight.

Note. The position of ribosomal RNA in Nycodenz gradients is always determined by ethidium bromide staining of ribosomal RNA in agarose gels instead of absorbance tracings [4,7-11]. The fractions of Nycodenz gradients are smaller than those of sucrose gradients and we do not collect the factions onto guanidine thiocyanate. Since the pellets of Nycodenz gradients do not contain mRNA, there is no need to extract RNA from the pellet.

*Extracting RNA from sucrose and Nycodenz gradients fractions*

1. Centrifuge the isopropanol precipitated gradient fractions for 15 min at top speed in a microcentrifuge for 15 minutes at 25^o^C. The tubes should be centrifuged with the hinges pointed out so the locations of the pellets in the tubes are known. Examine the tubes carefully by eye. Many of the tubes will show blue pellets at the bottoms of the tubes on the sides with the hinges, but others will not. You are not crazy. The tubes without blue pellets contain fractions with large amounts of protein (ie., fractions at the top of the sucrose gradients and fractions ~13-17 of Nycodenz gradients). The large amounts of protein make the pellets stick to the walls of the centrifuge tube so they do not slide to the bottom during centrifugation. Precipitation with ethanol produces pellets that tend to stick to the walls of the tubes more than do those that are precipitated with isopropanol.

2. Remove all of the isopropanol supernatant with a blue tip, being careful not touch the side of the tubes with the hinges. Before discarding the supernatant, hold the pipette tip up to the light and examine the contents carefully to make sure that it contains no pieces of pellet.

3. Remove the remainder of the ethanol supernatant with a yellow tip after centrifuging ~5 sec to transfer all of the liquid to the bottom of the centrifuge tube.

4. Add 180 μl DEPC-treated 75% ethanol to each tube, vortex, and centrifuge for 2 min to collect the pellets. Remove all of the ethanol, and air dry the pellets with the caps open. If all of the alcohol has been removed the pellets will dry completely in <15 min at 25^o^C.

5. Add 200 µl HNM containing 0.5% SDS and 100 µg/ml proteinase K to each tube. Vortex at moderate speed, and incubate for 1 hr at 50^o^C. During this period vortex several times to disperse the pellet, and be sure to wash the upper surfaces of the tubes with proteinase K digestion buffer to make sure that all of the precipitate is digested.

6. Add 200 µl phenol:cholorform:isoamyl alcohol (24:24:1) to each tube. Vortex hard several times over a period of several minutes. Centrifuge 1 min at top speed in a microcentrifuge. Inspect the tubes. An interphase should not be present. Stick a yellow tip through the aqueous phase into the organic phase, and remove all of the organic phase being careful to remove negligible amounts of aqueous phase which are seen as small bubbles that float to the surface of the organic phase in the pipette tip.

7. Add 20 µl 5 M NaCl and 550 µl ethanol to each tube, vortex and place at -20^o^C overnight.

8. Collect the pellets by centrifuging at top speed in a microcentrifuge for 15 minutes. Remove all of the supernatant in two steps as above: first remove ~775 µl ethanol with a blue tip, centrifuge the tubes 5 sec, and remove all of the remaining ethanol with a yellow tip being careful not to lose any pellet.

9. Wash the pellets by adding 180 μl DEPC-treated 75% ethanol to each tube, vortexing, and centrifuging for 2 min to collect the pellets. Remove all of the ethanol, vortex and dry with the lids open.

10. Examine the tubes carefully: every tube should contain a blue pellet of equal size at the bottom of the conical region. Dry the pellets with the tubes open at 25^o^C. This will not take more than 10 min if the ethanol has been completely removed.

11. Dissolve the pellets in 50 µl DEPC H_2_O by heating at 70^o^C for ten minutes and vortexing repeatedly as needed. Examine the tubes carefully. RNA that has not been completely dissolved appears as globules on the side of the centrifuge tube.

Notes:

1. The RNAs prepared by these procedures can be analyzed by Northern blots or RT-qPCR.

2. The conditions for alcohol precipitation of glycogen azure and nucleic acids differ. Ethanol and isopropanol precipitate glycogen azure in the absence of salt, but precipitation of DNA and RNA requires a final ~0.5 M NaCl concentration to neutralize the phosphates in the nucleic acid backbone.

3. The amount of ribosomal RNA in the peak fraction in Nycodenz gradients is high, usually fraction 4 or 5, and can be very difficult to dissolve.

4. We have used several extraction procedures during the past 15 years and some problems with alternative procedures should be noted. Extracting the sucrose gradient fractions with phenol:chloroform (1:1) is problematic because the combined density of dense sucrose gradient fractions and guanidine can be greater than that of the organic phase. This produces a confusing situation in which the organic phase is less and more dense, respectively, than the sucrose gradient fractions from the bottom and top of the gradient. Even worse, the organic and aqueous phases from the middle of the gradient do not separate. The density of the sucrose gradient fractions can be decreased by diluting each fraction with 0.2 ml HNM, but it is easier to avoid the problem in the first place by isopropanol precipitation. These problems are aggravated by the Trizol reagent which has a low ratio of chloroform to phenol.

*Developmental studies of mRNA translation*

Developmental changes in polysomal loading mRNA translation can be analyzed by with sucrose and Nycodenz gradients of purified spermatogenic cells from adult mice or staged prepubertal mice. We currently do not analyze purified spermatogenic cells because we do not know how to consistently prepare purified cell populations that are reliably free of the polysome breakdown that is documented in Figure 2B.

# Phase contrast examination of squashes of seminiferous tubules from 21 day and 22 day old mice reveal that the most advanced spermatids are step 4 and 5, respectively, in both CD-1 and C57/J6 black mice [4,6]. These stages are consistent with a report that early spermatids are first observed in 18 dpp testis and the duration of the stages of the seminiferous cycle [12,13]. Be sure to demand that the commercial supplier provides prepubertal mice of as exact an age as possible because the supplier may ship mice of an approximate age on the grounds that it “usually doesn’t make a difference”. One problem with staged prepubertal mice is that a small proportion of testicular cells have reached the most advanced stage, which results in low mRNA levels. This problem can be overcome by increasing the number of testes in the extract. For example, we are unable to quantify the distribution of the *Smcp* mRNA in sucrose and Nycodenz gradients by phosphorimage analysis of Northern blots of gradients containing an extract of the testes from one 21 dpp prepubertal mouse, we can do so easily with 10 testes from five mice.

*Using transgenic mice efficiently*

Each transgenic mouse is the end product of much time and energy and needs to be analyzed efficiently. One testis from an adult transgenic mouse can be used for prepare paraffin sections for immunohistochemistry or seminiferous tubule squashes and the other for Nycodenz or sucrose gradient anslysis. Alternatively, one can prepare a cytoplasmic extract of both testes in 640 μl HNM with 0.5% Triton X-100, sediment 250

μl on a sucrose gradient, while retaining the remainder of the extract in ice in a cold room, and sedimenting another 250 μl on a Nycodenz gradient after the sucrose gradient is complete.

**References**

1.Merrick WC, Hensold JO: **Analysis of eukaryotic translation in purified and semipurified systems.** *Curr Protoc Cell Biol Chapter* 2001, **11:**Unit 11.9.

2. Meyuhas O, Bibrerman Y, Pierandrei-Amaldi P, Amaldi F: **Analysis of polysomal mRNA.** *A Laboratory Guide to RNA: Isolation, Analysis, and Synthesis.* Edited by Krieg PA: New York, NY, Wiley-Liss: 1996: 65-104.

3. Rickwood, D: *Preparative Centrifugation. A Practical Approach, 2nd edn*., New York City, NY: Oxford Press; 1992.

4. Bagarova J, Chowdhury T, Kimura M, Kleene KC: **Identification of elements in the Smcp 5’ and 3’ UTR that repress translation and promote the formation of heavy inactive mRNPs in spermatids by analysis of mutations in transgenic mice.** *Reproduction 1010,* Sep 27. [Epub ahead of print].

5. Cataldo L, Mastrangelo MA, Kleene KC: **A quantitative sucrose gradient analysis of the translational activity of 18 mRNA species in testes from adult mice.** *Mol Hum Reprod* 1999, **5**:206-213.

6. Hawthorne SK, Busanelli RR, Kleene KC: **The 5’UTR and 3’UTR of the sperm mitochondria-associated cysteine-rich protein mRNA regulate translation in spermatids by multiple mechanisms in transgenic mice.** *Dev Biol* 2006, **297:**118-126.

7. Herbert TP, Hecht NB: **The mouse Y-box protein, MSY2, is associated with a**

**kinase on non-polysomal mouse testicular mRNAs.** *Nucleic Acids Res* 1999, **27**:1747-1753.

8. Antic D, Keene JD: **Messenger ribonucleoprotein complexes containing human ELAV proteins: interactions with cytoskeleton and translational apparatus.** *J Cell Sci* 1998, **111**:183-197.

9. Stenina OI, Shaneyfelt KM, DiCorleto PE: **Thrombin induces the release of the Y-box protein dbpB from mRNA: a mechanism of transcriptional activation.** *Proc Natl Acad Sci USA* 2001, **98:**7277-7282.

10. Tafuri SR, Wolffe AP: **Selective recruitment of masked maternal mRNA from messenger ribonucleoprotein particles containing FRGY2 (mRNP4).** J Biol Chem 1993: **268**:24255-24261.

11. Han S, Xie W, Hammes SR, DeJong J: **Expression of the germ cell-specific transcription factor ALF in Xenopus oocytes compensates for translational** inactivation of the somatic factor TFIIA. *J Biol Chem* 2003: **278**:45586-45593.

12. Bellvé AR, Cavicchia JC, Millette CF, O'Brien DA, Bhatnagar YM, Dym M:

**Spermatogenic cells of the prepuberal mouse. Isolation and morphological**

**characterization.** *J Cell Biol* 2005, **74**:68-85.

13. Russell LD, [Hikim](http://www.amazon.ca/exec/obidos/search-handle-url?_encoding=UTF8&search-type=ss&index=books-ca&field-author=Amiya P. Sinha Hikim) APS, [Ettlin](http://www.amazon.ca/exec/obidos/search-handle-url?_encoding=UTF8&search-type=ss&index=books-ca&field-author=Robert Ettlin) R, Clegg ED: *Histological and Histopathological Evaluation of the Testis.* Clearwater, FI: Cache River Press; 1990.

Additional file 2, Figure S1. Equipment for pouring sucrose gradients. The equipment is labeled and the numbers identify the components of the path through which the sucrose gradient solution flows: (1) Tubing connected to 18 gauge syringe needle on the gradient former; (2) the tubing extends through the peristaltic pump, and ends (3) in a capillary pipette in the ultracentrifuge tube. As illustrated, the ultracentrifuge tube is held manually, tilted slightly, with the tip of the pipette touching the wall of the centrifuge tube several mm above the meniscus, so the solution flows down the side of the tube onto the top of the gradient.

Additional file 3, Figure S2. Equipment for collecting fractions from sucrose and Nycodenz gradients. Panel A. Collecting fractions and obtaining an absorbance tracing from sucrose gradients. The equipment is labeled and the numbers identify the path of the liquid during fractionation. (1) A full-length 50 µl capillary tube inserted to the bottom of the ultracentrifuge tube in the bucket from the SW60Ti rotor. (2) A 7 cm piece of tubing connects the capillary tube to the inlet tubing of the UV analyzer. (3) A 60 cm piece of tubing is attached to the outlet tubing of the UV analyzer. This tubing extends through the peristaltic pump, and ends (4) in a short piece of glass capillary tubing which is used to collect the gradient into 1.5 ml microfuge tubes containing 0.32 g guanidine thiocyanate. Fractions are collected manually, and the size of the fractions is determined by lines drawn on the sides of 1.5 ml tubes. Panel B. Collecting fractions from Nycodenz gradients. (1) A full-length 50 µl capillary tube inserted to the bottom of the ultracentrifuge tube in the bucket from the SW60Ti rotor. (2) A 60-cm piece of tubing extends from the capillary pipette through the peristaltic pump, ending (3) in a short piece of glass capillary tubing which is used to collect fractions in 1.5 ml tubes. Fractions are collected manually, and the size of the fractions is determined by lines drawn on the sides of 1.5 ml tubes.

Additional file 4, Figure S3. Changes in absorbance during the analysis of a sucrose

gradient. A cytoplasmic extract from a pair testes from a 21 dpp male was prepared and sedimented on a sucrose gradient as described in Additional file 1. The changes in the absorbance tracing are identified as follows. Before beginning the tracing shown here, the tubing and flow cell were rinsed with FPD-DH_2_O and 0.1% SDS and FPD-DH_2_O with the absorbance of the UV analyzer set to 0.32. (1) With pump off the long capillary pipette is inserted into a 14 ml tube containing 40% sucrose and the pump is turned on. The absorbance increases sharply when the 40% sucrose enters flow cell due to Schlieren and returns to baseline when mixing ceases. (2) The pump is turned off, leaving the chart recorder on, and the long capillary pipette is inserted into the ultracentrifuge tube and the pump is restarted. (3) The absorbance starts to increase when polysomes at bottom of gradient begin to enter the flow cell, although this increase is sometimes preceded by a slight unexplained increase as soon as the pump is turned on. (4) Start collecting the fractions when the chart has advanced the distance calibrated as described in Additional file 1. (5) The absorbance increases dramatically as the lysis buffer at the top of the gradient which contains Triton X-100 enters the flow cell. When the line reaches the top of the chart the absorbance on the UV analyzer is set to 2.56. (6) The absorbance decreases dramatically when the top of the gradient exits the flow cell and is replaced by air. (7) The gradient ceases flowing into the final collection tube. The positions of the 10 fractions on the absorbance tracing that contain RNA to be extracted are numbered, and the peaks on the absorbance tracing representing polysomes containing 5 ribosomes, 80S single ribosomes, and the 60S ribosomal subunit are labeled.
