# Supplementary figures and images for: Quantitative analysis of mRNA translation in mammalian spermatogenic cells with sucrose and Nycodenz gradients
Source: Reprod Biol Endocrinol. 2010 Dec 25;8:155. doi: 10.1186/1477-7827-8-155 (PMC3022843; doi:10.1186/1477-7827-8-155)

## Slide 1
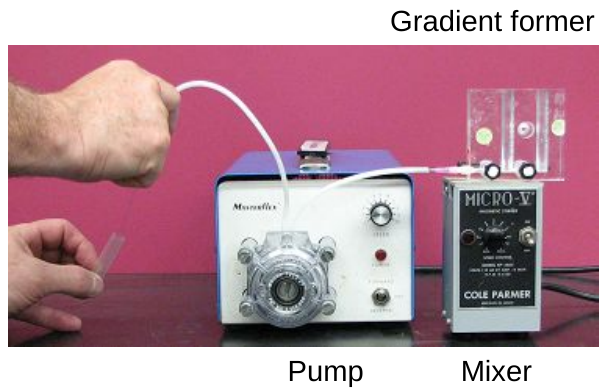

Gradient former
Pump
Mixer

Supplement: Additional file 2 — Figure S1: Equipment for pouring sucrose gradients. [file 1477-7827-8-155-S2.PPT]

## Slide 1
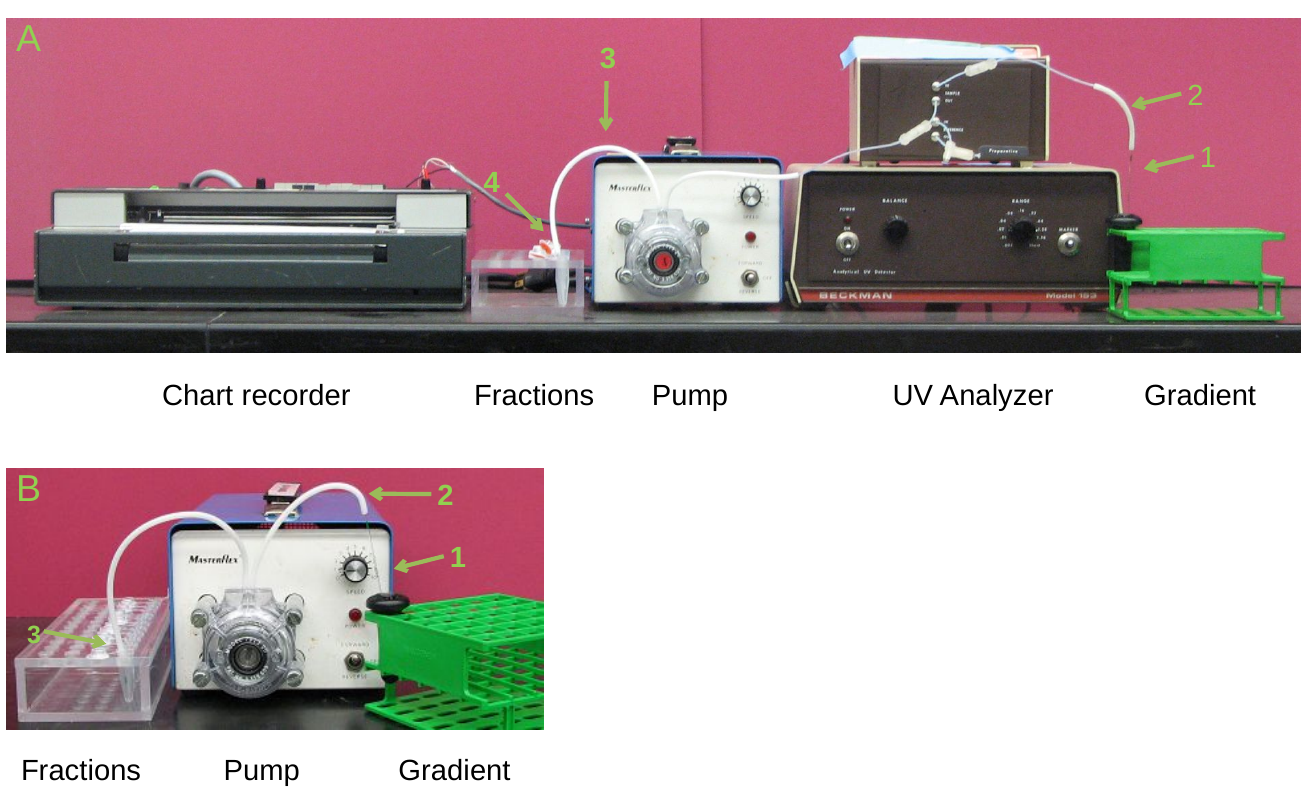

A
3
2
1
4
 Chart recorder Fractions Pump UV Analyzer Gradient
B
2
1
3
Fractions Pump Gradient

Supplement: Additional file 3 — Figure S2: Equipment for collecting fractions from sucrose and Nycodenz gradients. [file 1477-7827-8-155-S3.PPT]

## Slide 1
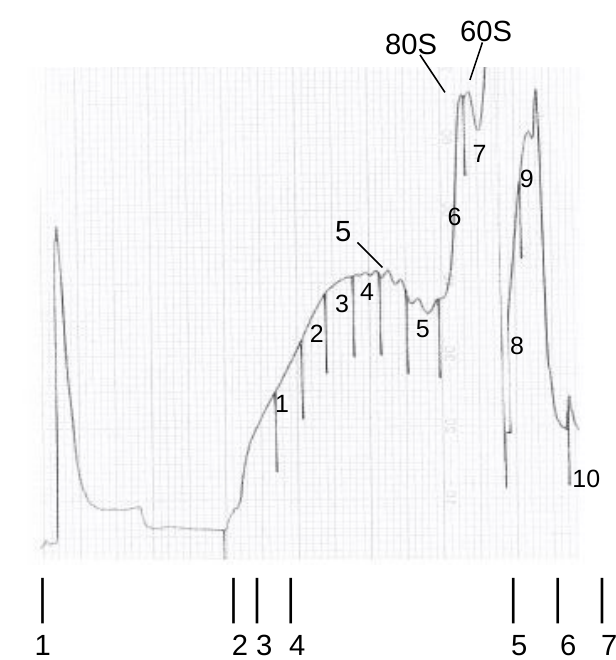

60S
80S
7
9
6
5
4
3
5
2
8
1
10
 │ ││ │ │ │ │
 1 2 3 4 5 6 7

Supplement: Additional file 4 — Figure S3: Changes in absorbance during the analysis of a sucrose gradient. [file 1477-7827-8-155-S4.PPT]
